# Investigating healthcare contacts of Dialysis patients by age and gender

**DOI:** 10.1186/s12913-019-3962-z

**Published:** 2019-02-27

**Authors:** James Todd, Adrian Gepp, Bruce Vanstone, Brent Richards

**Affiliations:** 1 Bond Business School, Bond University, Robina, Queensland 4229 Australia; 2 Director Critical Care Research, Gold Coast University Hospital, Southport, Queensland Australia

**Keywords:** Renal failure, Dialysis, Patient journey, Markov model, Patient demographics

## Abstract

**Background:**

The objective of this paper is to utilise a clinical costing system to investigate differences in the patient journey, defined as the sequence and timing of contacts with the Gold Coast Hospital and Health Services (GCHHS), for four dialysis patient groups defined based on age and gender. It is hypothesised that frequency of contact and form of contact will differ based on both gender and age.

**Methods:**

Data were provided for 393 patients discharged from the GCHHS facility with dialysis treatment between the 1st of January 2015 and the 31st of December 2016. Features extracted from the data included the number and type of contacts (inpatient admissions, outpatient appointments, and emergency department presentations), the likelihood of subsequent contact types, and time spent in and between contact types. Likelihoods of subsequent contact types were estimated by treating the sequence of contacts observed for each patient as a Markov chain and estimating transition probabilities.

**Results:**

Differences in patient journey were most prominent when considering age differences, with older patients being characterised by a greater volume of average contacts over the two-year period. The larger volume of average contacts was attributable to shorter times between all types of contacts with the GCHHS as well as an increased volume of inpatient admissions for older patients. Patient journeys did not consistently differ by gender, though some isolated differences were noted for older female patients relative to older male patients.

**Conclusions:**

Different patient groups are characterised by different patient journeys, and better understanding these differences will facilitate improved management of the resources required to service these patients. Clinical costing systems represent a valuable and easily accessible source of data for formulating institution-specific expectations of healthcare utilisation for different groups.

## Background

The increasing number of patients requiring continuing kidney dialysis treatment, along with other renal replacement therapies, has been historically driven by factors such as an aging population and increases in the prevalence of diabetes [1–3]. Though the incidence of dialysis patients has stabilised in recent years, past increases in patients in this area of the health system, coupled with the relatively high cost of dialysis [4], has motivated research into various aspects of patient treatment, management and experience.

Research addressing the need to effectively manage dialysis patients has looked at the forecasting of resource requirements and the cost-effectiveness of treatment options. The effective managing of resources through forecasting demand has been of particular importance given the long-term involvement of dialysis patients in the health care system. Research focusing on this aspect of patient management has been a historical area of interest, with Davies R., Johnson D., and Farrow S. [5] using a Markov-chain model in 1975 to model the treatment programme for dialysis patients. Resources considered included dialysis machines, bed stations, pathology facilities, and staff. Even in more contemporary settings resource restrictions continue to be relevant, motivating research into the cost-effectiveness of treatment, such as by Howard K. et al. [3] and Carnero M. C. and Gómez A. [6]. Howard K. et al. [3] investigated differences in financial costs and quality adjusted life years associated with different renal therapy modalities, while Carnero M. C. and Gómez A. [6] considered a multicriteria decision-model approach for selecting combinations of maintenance policies for dialysis subsystems in hospitals.

A common aspect of many studies in this area is the acknowledgement of the potential differences in results between patient groups. While differences in patient experience based on demographic factors specifically have been noted in several studies, these differences have rarely been the primary focus. This is despite the increasing emphasis placed on shared decision-making for patients and physicians based on information which is appropriately personalised [7]. Though one study identified by the authors does aim to consider differential outcomes and quality of life for dialysis patients according to a wide range of demographic factors [8], it has not yet been published. This study is explicitly motivated by the need for greater information for all stakeholders but collection of data with longitudinal survey methods does not allow for immediate results.

The present study similarly aims to explicitly investigate differences in patient experience based on demographic features. It differs from that proposed by Walker R. et al. [8], however, in that the primary focus is on patient contact with health services and that the data utilised is from an existing database which should be present in some form for all healthcare providers. These two key aspects reflect the two purposes of this paper. The first purpose is to provide more information about how different groups of dialysis patients engage with health services. Previous research on dialysis patients has demonstrated that age and treatment modality are associated with differences in outcomes and quality of life [2] and demographic features are associated with differential utilisation of healthcare services [9]. To the best of the authors’ knowledge, however, research has not quantified the differences for the frequency and primary types of health services contact. While the significance of differences in utilisation have been established, such as greater use by older patients, this is insufficient on its own to be used for planning purposes. Within the context of planning, it is also important to investigate how these differences materialise, such as in the expected number of contacts and types of contact for different groups. The first goal of this article is to illustrate an approach for healthcare providers to better address this issue. Differences in the interaction of patients with health services may have important implications from a management perspective if these demographic features could be used to formulate expectations of a patient’s experience early. The second, related purpose is to demonstrate how clinical costing systems can be better leveraged to allow healthcare providers to extract institution-specific insights about the patients they service. All healthcare providers are expected to maintain some form of clinical costing system, and this serves as an underutilised source of data that does not require additional expenditure or further data collection to use. Additionally, the data are directly relevant for use in planning and resource allocation as it is institution-specific.

This paper aims to use clinical costing information for services provided to patients to assess whether differences in engagement with health services can be identified for four major patient groups defined based on age and gender. The greater use of health services by older patients has been well documented and it is expected to be a pattern which will remain consistent in this context. Consideration of gender is motivated by many related studies [e.g. 2,6,11] controlling for it. While the expectation is not as clear as for age, gender remains an easily accessible demographic factor and worth considering, especially given the increasing emphasis on personalising information for patients. Thus, it is hypothesised that frequency of contact and form of contact will differ based on both gender and age.

## Methods

### Data used

Data for this study were provided by the Australian Gold Coast Hospital and Health Services (GCHHS). The data were extracted on 2017/11/08 from the GCHHS’s clinical costing system and consisted of all patients discharged from the GCHHS facility with either haemodialysis or peritoneal dialysis between 2015/01/01 and 2016/12/31. To address privacy concerns the data had all identifying information removed prior to being provided and included 393 unique patients. The data was provided in the form of an Excel document consisting of several sheets of information, which could be linked through patient and encounter IDs. The relevant sheets are summarised in Table 1.

**Table 1:** Data Provided

Excel Sheet	Rows	Unique Patients
Inpatient Admissions (I)	40,416	393
Outpatient Appointments (O)	10,144	272
Emergency Department Presentations (E)	875	231

### Outcomes for each patient were not provided

A patient’s journey in the context of this paper is the sequence and timing of GCHHS contact events, with three types of contacts being possible. These contact types are inpatient admissions, outpatient appointments and emergency department presentations. An inpatient admission is the scenario in which a patient is admitted to the hospital and is most often the result of emergency department encounters, but can also be from outpatient appointments, a dialysis episode or home. Outpatient appointments involve contact with a clinic without hospital admission, such as consultation appointments. They include only clinic encounters, and do not include radiology or pathology diagnostic encounters. Throughout the results presented in this report, these three contact types are coded as “I”, “O”, and “E” respectively.

This study investigates differences in the patient journeys for four major patient groups defined using binary splits of age and gender. The splitting value selected for age was 65 years old, with all patients younger than 65 being classified as “Young”, and all other patients being classified as “Old”. This age was selected because it is close to the median age of patients at their first recorded contact (64), and because it has a real-world translation as the Australian retirement age. It is also the splitting age employed by Walker R. et al. [8] in their current study considering dialysis outcomes. The split of patients across these groups is shown in Table 2.

**Table 2:** Membership of Patient Groups

	Male	Female	Totals
*Young*	127	81	208
*Old*	130	55	185
*Totals*	257	136	393

These patient groups are subsequently referred to using the first letter of their gender and age classification. For example, patients who are male and at least 65 years old are referred to as the patient group MO. When results are presented for all patients, this aggregated group is referred to as AP.

### The patient journey

The aspects of patient journey investigated in this study are:

- Number of GCHHS contacts and proportion of each type
- Likelihood of each contact type conditional on the previous contact type
- Average time of each contact type and average time between contacts
- Average time between contacts conditional on previous contact type

Each aspect of the patient journey was assessed for the four defined patient groups as well as the aggregate of all four.

Calculation of the number and proportion of each contact type across each patient grouping is performed because it provides a simple way to assess differences in the volume and type of contacts for each group. These high-level differences are important to identify, while more detailed differences regarding waiting times and the likelihood of different contact types require a more involved approach. Pearson’s Chi-Squared test was used to assess whether the proportion of contacts for each type (I, O and E) vary according to the four gender and age subgroups.

For calculation of likelihoods, a Markov chain model was used. While the process followed is not a Markov chain in that a patient does not remain in a single state before moving to a new state, the transition probabilities can still be estimated in the same way. The only change is to the interpretation of these probabilities, as they are no longer the probability of transitioning to a given state in each unit of time. They are instead interpreted as the likelihood of the next contact being of a given type, conditional on the previous contact type.

Average time in each contact type is calculated using the difference in admission and discharge times. In the case of outpatient appointments, only the starting time was available, and so the duration of each was considered to be 1 hour in all cases. This is because outpatient appointments are relatively short, and a reasonable value was needed to ensure that time in a fourth state, in which a patient was not in any form of contact with a health services, did not continue across outpatient appointments. The waiting time in this fourth state represents the time between presentations to the GCHHS.

The final aspect of patient journey assessed was the average time between contacts, conditional on the previous contact type. As for average waiting times, this is simply equal to the average waiting time in the fourth state which represents no contact with the hospital. The only modification is that it is stratified according to which of the three possible contact types preceded the state.

The statistical significance of differences in waiting times (both in each contact and between contacts) are tested by evaluating the Analysis of Variance (ANOVA) for a linear regression model of waiting time (dependent variable) based on the independent variables of contact type (I, E and O), the age and gender grouping (MY, MO, FY, FO) as well as all interaction terms. Note that if the interaction terms are not statistically significant then the regression is estimated without them. An alternative approach would have been to simply compare the average waiting times, but this approach was not preferred as it ignores the full information available about each waiting time. Consequently, the regression-based approach is more reliable.

All data manipulation and calculations were performed using the statistical software R version 3.3.1 [10].

## Results

The findings of this study are presented in the following sub-sections relating to the four aspects of patient journey investigated.

### Number and average of GCHHS contacts

Tables 3, 4, and 5 display the volume and type of contacts for each of the four patient groups considered, as well as for all patients considered together. Table 3 presents the counts of each contact type by group, while Table 4 presents the average number of each contact type per patient within each group. Finally, Table 5 presents the proportion of each contact type in the average patient journey for each patient group. All numbers are rounded to two decimal places.

**Table 3:** Contact Frequencies by Type

	AP	MY	MO	FY	FO
Inpatient	40,416	10,171	17,422	5417	7406
Outpatient	10,144	3489	3637	1976	1042
Emergency	875	302	294	163	116
All	51,435	13,962	21,353	7556	8564

*AP* All Patients, *MY* Male & Young, *MO* Male & Old, *FY* Female & Young, *FO* Female & Old

**Table 4:** Average Number of Contacts by Type

	AP	MY	MO	FY	FO
Inpatient	102.84	80.09	134.02	66.88	134.56
Outpatient	25.81	27.47	27.98	24.4	18.95
Emergency	2.23	2.38	2.26	2.01	2.11
All	130.88	109.94	164.25	93.28	155.71

*AP* All Patients, *MY* Male & Young, *MO* Male & Old, *FY* Female & Young, *FO* Female & Old

**Table 5:** Average Proportion of Contacts by Type

	AP	MY	MO	FY	FO
Inpatient	78.58%	72.85%	81.60%	71.70%	86.47%
Outpatient	19.72%	24.99%	17.04%	26.16%	12.17%
Emergency	1.70%	2.16%	1.38%	2.15%	1.36%
All	100.00%	100.00%	100.00%	100.00%	100.00%

*AP* All Patients, *MY* Male & Young, *MO* Male & Old, *FY* Female & Young, *FO* Female & Old

From the three tables presented here, several differences in the number of contacts and the distribution of contact types can be identified. Younger patients, under the age of 65 at the time of their first contact, tend to have a much smaller number of contacts with an average of at least 50 fewer total contacts than their older counterparts for both male and female patients. The differences in the number of contacts can be attributed primarily to the large reduction in the number of inpatient admissions for younger patients, with outpatient appointments and emergency department presentations being of comparable frequency between age groups. Because of the smaller number of inpatient appointments for younger patients, the relative proportion of other contact types for the average patient journey in the younger groups is larger, as shown in Table 5. Differences on other characteristics are less dramatic, with the most important being the notably lower number and proportion of outpatient appointments for patients who are female and older than 65. This difference is notable because younger female patients do not appear to differ greatly from younger male patients in terms of outpatient appointments, making this gender difference specific to the older patient groups.

Overall, differences were statistically significant at the 1% level (Pearson’s Chi-Squared test statistic = 923.56, *P* < 0.001). This demonstrates that the proportion of contact types (I, O and E) does depend on age and gender groupings. Furthermore, repeated application of the Pearson’s Chi-Squared test on each possible pair of groups revealed all pairwise differences to be statistically significant at the 1% level except differences between MY and FY patients that are not even significant at the 10% level. This illustrates the source of the overall differences. In this case, age differences are relevant for both genders, while gender differences only appear relevant for older patients.

### Likelihood of contact type

**Fig. 1:**
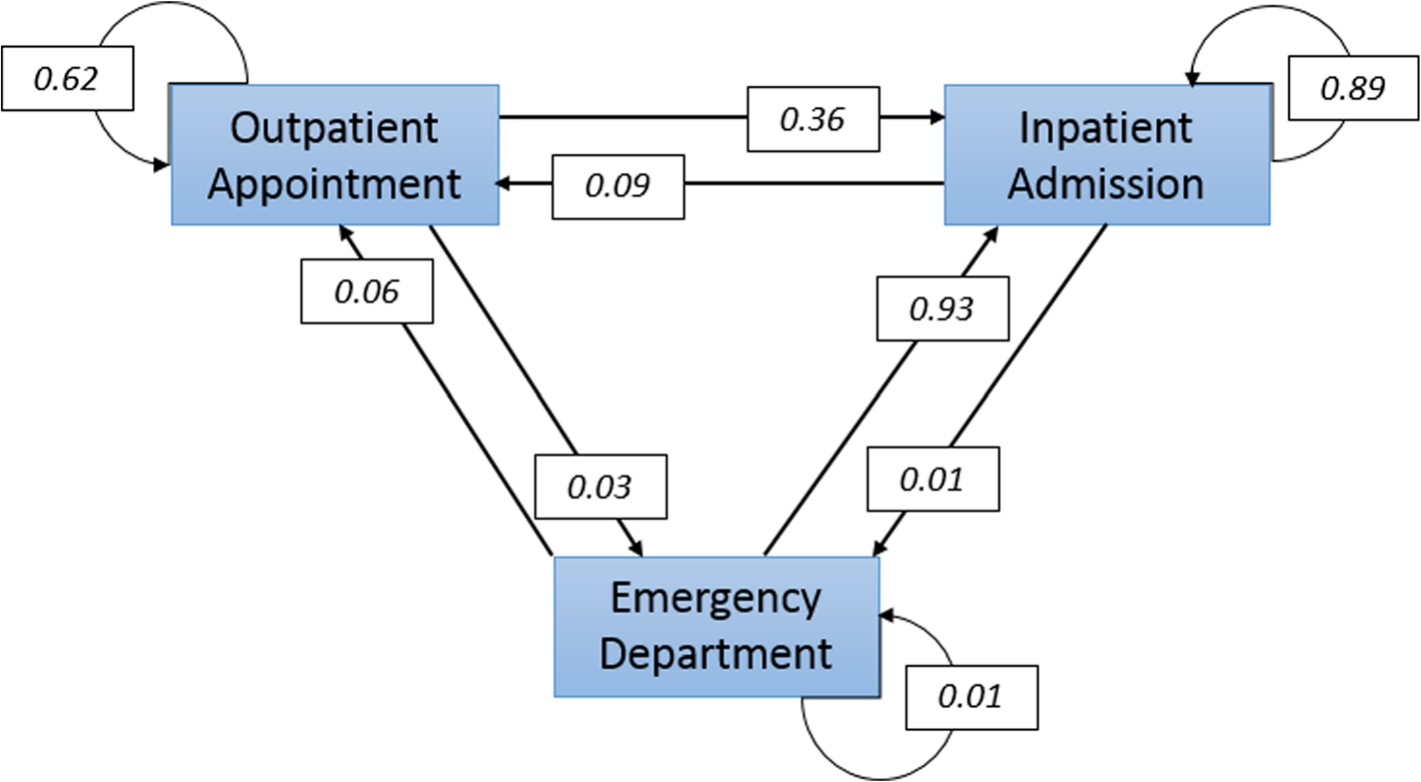
Next Contact Likelihoods (All Patients)

The likelihood of the next contact type, dependent only on the previous contact type, was estimated by treating the sequences of patient contacts as a Markov chain. The transition matrices for each patient group were estimated, with transition probabilities being the likelihood of the next contact type rather than movement from one state to another. Figure 1 shows a graphical representation of the transition probabilities estimated for all patients, but to facilitate comparisons the transition probabilities for each patient group were summarised in Table 6. The first column indicates the previous contact type and the second column indicates the next contact type to which the probabilities presented in all other columns relate.

**Table 6:** Next Contact Likelihoods (All Groups)

Previous Contact	Next Contact	AP	MY	MO	FY	FO
*E*	*E*	1.49%	3.31%	0.34%	1.23%	0.00%
*E*	*I*	93.00%	89.74%	96.58%	92.02%	93.86%
*E*	*O*	5.51%	6.95%	3.08%	6.75%	6.14%
*I*	*E*	1.41%	1.83%	1.09%	1.96%	1.18%
*I*	*I*	89.18%	86.28%	90.33%	86.41%	92.46%
*I*	*O*	9.41%	11.89%	8.58%	11.63%	6.36%
*O*	*E*	2.67%	2.80%	2.66%	2.61%	2.42%
*O*	*I*	35.59%	32.61%	39.20%	29.82%	43.85%
*O*	*O*	61.74%	64.59%	58.14%	67.57%	53.73%

*AP* All Patients, *MY* Male & Young, *MO* Male & Old, *FY* Female & Young, *FO* Female & Old, *E* Emergency Department Presentation, *I* Inpatient Admission, *O* Outpatient Appointment

Inspecting Table 6, the likelihood of sequential outpatient appointments for patients who are female and at least 65 years old is notably lower compared to that of other patient groups. This is consistent with the earlier observation that this patient group is characterised by a lower proportion and average number of outpatient appointments per patient. The lower likelihood of sequential outpatient appointments suggests that even when these patients do have this type of contact they are more likely to return to the inpatient admission type to receive their dialysis treatment. It can also be seen that younger patients are more likely to have contact with the GCHHS through outpatient appointments and emergency department presentations, while older patients in general are more likely to have inpatient appointments.

While differences can be identified for patients within different age categories, no overarching differences in the likelihood of subsequent contact types appear to be associated with gender.

### Average time by contact type

The next element of patient journey presented is the average time spent in each contact type per contact, with times expressed in days. The three contact types are also supplemented by a fourth possible state “N”, defined as the state in which a patient is not in contact with the GCHHS. Thus, average time between contact types can be assessed for each patient group in addition to the average time spent within each. The empirical averages are displayed in Table 7.

**Table 7:** Average Time (in days) in each Contact Type

	AP	MY	MO	FY	FO
I	0.4072	0.4225	0.3987	0.4386	0.3861
O	0.0417	0.0417	0.0417	0.0417	0.0417
E	0.1595	0.1512	0.1703	0.1558	0.1586
N	2.9954	3.4587	2.6679	3.5059	2.6733

*AP* All Patients, *MY* Male & Young, *MO* Male & Old, *FY* Female & Young, *FO* Female & Old, *E* Emergency Department Presentation, *I* Inpatient Admission, *O* Outpatient Appointment, *N* No Contact

As mentioned previously, data relating to the end times for outpatient appointments were unavailable and so all such appointments are treated as lasting for 1 hour. The impact of this assumption is expected to be negligible, but the outpatient appointments are still displayed here to highlight the fact that this modification to the data was made.

Table 7 shows age appears to be a differentiator of patient experience, while gender is not associated with differences in this aspect of the patient journey. Younger patients have longer average stays as inpatient admissions and between contact types. While patients who are male and older than 65 also have slightly longer average times in emergency department presentations, the difference is relatively small and is not observed for patients who are female and older than 65.

The smaller average time between contact types for older patients, represented by state N, reflects the larger average number of contact types for these patients over the two-year period observed earlier in Table 4.

The Analysis of Variance for a linear regression revealed that the four subgroups (MY, MO, FY, FO) were found to influence the average waiting time both as a main effect (F value = 48.37, *P* < 0.001) and as an interaction effect (F value = 14.56, P < 0.001) with the contact type (E, O, I). This relationship was statistically significant at the 1% level as shown by the *p*-values and demonstrates that the time in each contact type does depend on age and gender groupings. The source of these differences was investigated further by separately analysing data for each pair of the MY, MO, FY and FO subgroups. Patient age was found to be statistically significant (*P* < 0.001 for both main and interactive effects) for both male patients (MY versus MO) and female patients (FY versus FO). On the other hand, gender was not found to be significant for either younger ages (MY versus FY) or older patients (MO versus FO). Although pairwise comparisons where both age and gender differ are less meaningful, we include them for completeness. Unsurprisingly, significant differences (*P* < 0.001 for both main and interactive effects) were also found between older male patients and younger female patients (MO versus FY) and younger male patients and older female patients (MY versus FO).

### Average time between contacts conditional on previous contact type

The final aspect of patient journey assessed is the average time in days after a specific contact type until any other contact type. These average times are presented in Table 8, with the first column denoting the specific contact type from which time is measured.

**Table 8:** Average Time (in days) Between Contacts Given Previous Type

	AP	MY	MO	FY	FO
I	2.1686	2.2946	2.1158	2.1919	2.1135
O	6.3742	6.8603	5.4942	6.7701	7.0854
E	1.7419	2.5646	0.5813	2.0865	1.5585

*AP* All Patients, *MY* Male & Young, *MO* Male & Old, *FY* Female & Young, *FO* Female & Old, *E* Emergency Department Presentation, *I* Inpatient Admission, *O* Outpatient Appointment

This table shows that the larger average times between contact events for older patients from Table 7 can be attributed in part to the lower number of outpatient appointment contact types for these patients. The average times from an outpatient appointment to another contact are larger than the average times from either inpatient admissions or emergency department presentations. Table 8 also shows a general trend towards shorter times between contacts of all types for older patients. The only exception is the time between contacts after an outpatient appointment for older female patients, which is greater than the average time between contacts in any other scenario.

The differences between all groups for average times following an emergency department presentation are interesting in that the differences are relatively large between all patient groups. Gender also appears to reverse in effect between the two age groups, with younger (older) female patients having shorter (longer) times until subsequent events than their male counterparts.

As was found for the time in each contact type, the time between each contact type was found to depend on age and gender groupings. The age and gender subgroups were again statistically significant at the 1% level in terms of both a main effect (*F* value = 10.46, *P* < 0.001) and an interaction effect with contact type (*F* value = 10.30, *P* < 0.001). Comparing each possible pair of subgroups found significant differences (*P* < 0.01 for both main and interaction effects) associated with age for male patients (MY versus MO) and with gender for older patients (MO versus FO). Additionally, a significant difference (*P* < 0.001 for both main and interaction effects) between older male patients and younger female patients was noted.

## Discussion

### Key findings

The aspects of patient journey for which results were presented highlight findings regarding the differences in the patient journey for different groups of dialysis patients. As hypothesised, several differences were observed between age groups for each aspect of the patient journey, but the same support was not found for the hypothesis that differences would be found based on gender. Several specific differences were observed between groups for each aspect of the patient journey, generally associated with patient age but not patient gender.

Older patients were characterised by a higher volume of average contacts with the GCHHS, driven by a larger average number of inpatient admissions for these patients. The tendency for older patients to have more frequent inpatient appointments was also evident when considering the likelihood of subsequent contact type dependent on previous contact type. Older patients were less likely to have their next contact type be an outpatient appointment regardless of their previous contact type. The large number of average contacts of older patients with the GCHHS over the two-year period considered was highlighted again in the shorter average time periods between contact events. The shorter times between contacts was found both in aggregate and when conditioning on specific contact types.

While the short average time between contacts is partially a feature of older patients having shorter times between events in nearly all instances, it is also a function of the increased likelihood of inpatient admissions for these patients. Inpatient admissions were shown to have shorter times until subsequent contact events were compared to outpatient appointments for every patient group considered.

In addition to differences between age groups, the patient group representing older female patients stood out from the older male patients group in a few regards despite no consistent gender differences being noted for younger patients. In particular, the differences observed between older and younger patients were more pronounced for older female patients when considering the proportion of outpatient appointments and the likelihood of subsequent contact types. This patient group had a lower proportion of outpatient appointments than its male counterpart. Further, after an outpatient appointment, the probability of a subsequent contact type being an inpatient admission was higher, while it was lower for another outpatient appointment.

### Implications

The results of this paper have several implications from both management and patient perspectives. The differences in patient journey observed here indicate that demographic information on patients requiring dialysis could be useful in forecasting the demand on the resources required to service these patients, such as dialysis machines, beds, and staff. Being better able to budget for the specific resources which are required for different patients and the overall demand allows for more efficient allocation of these resources and thus better utilisation. For example, having many older patients who require dialysis would be expected to be associated with a large demand on the resources associated with inpatient admissions, with a relatively high frequency of admissions for each patient relative to what would be expected from younger patients. By quantifying these differences, management could endeavour to match the availability of resources with the flow of admissions. For example, identifying periods of high demand in advance means appropriate actions can be taken to ensure that the requisite resources to meet that demand are made available. Similarly, information regarding the relationship between patient demographics and patient journey can be used to monitor patient progression over time. In this manner, management of these patients can be a continuing process.

Associating demographic features with changes in the patient journey will also better allow for the identification of specific patient groups where process improvement is required. Changes to existing processes and methods of catering to these patients can then be tailored to the specific group, rather than adopting changes applicable to all patients. For example, if costly patient groups could be incentivised to use lower-cost contact types without compromising treatment effectiveness, health facilities would be better able to direct savings towards other patients or areas to improve outcomes.

Finally, from the patient perspective, by better understanding the patient journey and how it varies for different demographics, patients can be better informed as to what to expect for their own experience.

### Limitations

Several limitations for this study should be considered when interpreting the results. First, data for patients from the GCHHS were used, and the characteristics of these patients may not fully reflect the characteristics of dialysis patients at other hospitals or in other regions. However, the intention of the paper is to illustrate a method which can be applied by other institutions with similar data sources to derive results specific to their patient populations rather than generalise those shown here. Secondly, all data provided related to contact with the GCHHS between 2015/01/01 and 2016/12/31 for patients who were receiving dialysis over this period. This means that no data were available that explicitly detailed the starting or ending points for a patient’s dialysis therapy. Thirdly, no data were available detailing the time at which outpatient appointments ended, only the starting time of each appointment. This necessitated the use of a constant duration of 1 hour for outpatient appointments for calculating average time within and between contact types. Finally, no data was available from the clinical costing system detailing the existence of comorbidities or the cause of kidney failure, which ideally would have been considered in this type of analysis as well. Demographic data was also limited to the age and sex of patients. This is a limitation of the retrospective nature of the data source considered. For most treatment facilities this data are easily and quickly accessible but may not be as comprehensive as that collected in a prospective study (for example, a survey). This limitation could reasonably be expected to be overcome by healthcare providers performing similar analysis for their own serviced populations by leveraging other data sources to supplement that available in costing systems.

## Conclusion

This study investigated aspects of the patient journey for four groupings of dialysis patients defined using age and gender. Several differences in patient journey, which was considered as the sequence and timing of different contact types with the GCHHS, were identified for age groups but not gender. Patients aged 65 or over demonstrated a much greater tendency to engage with the GCHHS through inpatient admissions and with a greater frequency than younger patients. While differential utilisation of healthcare services is not unexpected, the methods employed and data used make the quantification of these differences immediately relevant to the GCHHS. The findings of this study have several implications for improving management of resources and dialysis patients using demographic data to better formulate expectations of patient journey. In particular, this paper provides motivation for further research utilising data from clinical costing systems to derive real insights into patient experiences and for investigating differences in patient journey associated with demographic factors. The easily accessible and institution-specific nature of this data source means that analysis and outcomes do not require generalisation, with healthcare providers able to perform similar analysis to that described in this paper using their own data.

Future research should expand on the findings of this paper by incorporating more information detailing patient journeys. In particular, a more complete picture of the patient journey could be achieved by considering the exact starting and ending dates of dialysis therapy for each patient, rather than the first and last sessions within a two-year snapshot as considered in this paper. The eventual outcome for each patient should also be considered to assess the effect of differing contact types, frequencies, and patient groups in this respect. Finally, using more demographic information and larger populations would allow for the definition of more detailed patient groups and the consideration of the influence of other demographic factors on patient journey. This could be achieved by combining data from clinical costing systems with other existing sources of data maintained by healthcare providers.

## Abbreviations

AP: All Patients
E: Emergency Department Presentation
FO: Female, Old
FY: Female, Young
GCHHS: Gold Coast Hospital and Health Services
I: Inpatient Admission
MO: Male, Old
MY: Male, Young
N: No Contact
O: Outpatient Appointment

## References

[1] Orlando LA, Belasco EJ, Patel UD, Matchar DB (2011). The chronic kidney disease model: a general purpose model of disease progression and treatment. BMC Med Inform Decis Mak.

[2] Lukowsky LR, Mehrotra R, Kheifets L, Arah OA, Nissenson AR, Kalantar-Zadeh Prof K (2013). Comparing mortality of peritoneal and hemodialysis patients in the first 2 years of dialysis therapy: a marginal structural model analysis. Clin J Am Soc Nephrol.

[3] Howard K, Salkeld G, White S, McDonald S, Chadban S, Craig JC, Cass A (2009). The cost-effectiveness of increasing kidney transplantation and home-based dialysis. Nephrology.

[4] de Wit GA, Ramsteijn PG, de Charro FT (1998). Economic evaluation of end stage renal disease treatment. Health policy.

[5] Davies R, Johnson D, Farrow S (1975). Planning patient care with a Markov model. Oper Res Q.

[6] Carnero MC, Gómez A (2016). A multicriteria decision making approach applied to improving maintenance policies in healthcare organizations. BMC Med Inform Decis Mak.

[7] Churchill DN, Jassal SV (2014). Dialysis: destination or journey. J Am Soc Nephrol.

[8] Walker R, Derrett S, Campbell J, Marshall MR, Henderson A, Schollum J, Williams S, McNoe B (2013). Dialysis outcomes in those aged ≥65 years. BMC Nephrol.

[9] Levine R, Javalkar K, Nazareth M, Faldowski RA, de Ferris MD-G, Cohen S, Cuttance J, Hooper SR, Rak E (2018). Disparities in health literacy and healthcare utilization among adolescents and young adults with chronic or end-stage kidney disease. J Pediatr Nurs.

[10] R Core Team. R: a language and environment for statistical computing. Vienna: R: foundation for statistical Computing; 2016.

